# A Successful Rechallenge in a Patient With Oxaliplatin-Induced Tonic–Clonic Seizures Under Anticonvulsant Therapy

**DOI:** 10.1155/crom/4707066

**Published:** 2025-11-30

**Authors:** Mona Mirzaee, Behrad Azadmehr, Saeid Haji Aghajani, Zahra Jahangard-Rafsanjani

**Affiliations:** ^1^Department of Clinical Pharmacy, Tehran University of Medical Sciences (TUMS), Tehran, Iran; ^2^Department of Hematology-Oncology, Imam Khomeini Hospital Complex Cancer Institute, Tehran, Iran

**Keywords:** anticonvulsant, chemotherapy-related neurotoxicity, colorectal cancer, oxaliplatin, seizures

## Abstract

**Background:**

Oxaliplatin is an alkylating chemotherapeutic agent which is FDA-approved for colorectal cancer treatment. The most frequent neurologic complication reported with oxaliplatin is acute peripheral neuropathy. Posterior reversible encephalopathy syndrome (PRES) is another neurologic adverse effect that has been reported with oxaliplatin-based chemotherapy regimens resulting in seizures. Oxaliplatin-induced seizure in the absence of PRES is rare and has been reported in four case reports.

**Case Presentation:**

We report a case of tonic–clonic seizures after oxaliplatin administration in the absence of any other abnormal radiological or laboratory findings in a 58-year-old male diagnosed with rectal adenocarcinoma. In this case, tonic–clonic seizures occurred a few hours after oxaliplatin administration in two episodes lasting 1 min each, two and a half hours apart. The patient's vital signs, EEG, and brain MRI showed no abnormalities. The patient received levetiracetam after the seizure onset and was successfully retreated with oxaliplatin 6 months later.

**Conclusion:**

This case highlights a rare presentation of oxaliplatin-induced seizure occurring in the absence of PRES or other identifiable metabolic, structural, or infectious causes. Notably, successful rechallenge with oxaliplatin was achieved after a prolonged seizure-free interval and under antiepileptic coverage, suggesting that rechallenging may be considered in selected patients following multidisciplinary evaluation.

## 1. Introduction

Oxaliplatin is an alkylating chemotherapeutic agent, which consists of a platinum compound that binds to DNA and forms cross-links resulting in cell death by the inhibition of DNA replication, transcription, and holding up of the cell cycle. Oxaliplatin effectively acts on fast-growing tumors like those in the gastrointestinal system, with a high cell turnover rate [1, 2].

Oxaliplatin is FDA-approved in combination with 5-fluorouracil and leucovorin (FOLFOX regimen) and in combination with capecitabine (CAPOX regimen) in the adjunctive treatment of Stage III colorectal cancer after resection of the primary tumor and for the treatment of metastatic colorectal cancer [1, 3, 4].

The most adverse effects associated with oxaliplatin are gastrointestinal, hematologic, hepatic, and neurologic side effects. The most frequent neurologic complication reported with oxaliplatin is acute peripheral neuropathy [5]. Posterior reversible encephalopathy syndrome (PRES) is another neurologic adverse effect that has been reported with oxaliplatin-based chemotherapy regimens resulting in seizures [6]. Although oxaliplatin-induced seizures in the absence of PRES are rare, it has been mentioned in case reports [7]. We herein present another case of tonic–clonic seizures in the absence of PRES in a patient after receiving the first dose of oxaliplatin.

## 2. Case Presentation

A 58-year-old male with a recent diagnosis of Stage IIIb (T4a, N1, M0) proficient mismatch repair (pMMR) rectal adenocarcinoma in August 2024 was initiated on neoadjuvant chemoradiotherapy (CRT). The patient had no other past medical or drug history. The CRT regimen included 28 sessions of pelvic radiotherapy concomitant with oral capecitabine at a total daily dose of 2500 mg, administered in two divided doses (1500 mg in the morning and 1000 mg in the evening). Following completion of CRT, the patient was prescribed a CAPOX chemotherapy regimen comprising oxaliplatin 190 mg IV on Day 1 and oral capecitabine 1500 mg every 12 h starting from the evening of Day 1 until the morning of Day 15, repeated every 21 days, for a planned total of four cycles.

On the 1st day of the first course of chemotherapy, the patient received oxaliplatin in the morning. The patient received dexamethasone 8 mg IV, granisetron 3 mg IV, and chlorpheniramine 10 mg IV, as premedication, 30 min before oxaliplatin infusion. Approximately 10 h later, before taking capecitabine, he experienced a generalized tonic–clonic (GTC) seizure. The seizure was characterized by upward gaze, GTC movements, jaw locking, sialorrhea, and a postictal phase without urinary incontinence and lasted for approximately 1 min. The patient presented to the emergency department and experienced a second GTC seizure approximately 2.5 h after the initial episode which manifestations were identical to the first episode. He remained conscious and alert between the two events.

At the time of emergency admission, vital signs were stable: blood pressure 129/70 mmHg, heart rate 90 bpm, respiratory rate 18 breaths/min, temperature 37°C, and oxygen saturation 97% on ambient air. Neurological examination was normal. Laboratory findings, including complete blood count, comprehensive metabolic panel, cardiac troponin, and NT-proBNP, were within normal limits. Electroencephalography (EEG) showed an abnormal EEG due to generalized slowing of background suggestive of diffuse cerebral dysfunction but without epileptiform discharges. Noncontrast magnetic resonance imaging (MRI) of the brain showed no acute abnormalities or signs of PRES. The patient had no other risk factor suggestive of epileptic seizures, including family history, past febrile convulsion, intracranial infection, and/or head trauma.

The patient was treated with intravenous levetiracetam 3 g as a loading dose, followed by 1 g IV every 8 h for 3 days. After 3 days of hospitalization and at the time of discharge, the intravenous levetiracetam was switched to oral levetiracetam at a dose of 1 g three times a day. After 1 month, the dose was tapered to 500 mg every 8 h without recurrence of symptoms.

Considering the time interval between oxaliplatin administration and the occurrence of seizures, and the absence of structural or metabolic abnormalities, oxaliplatin was the most suspected cause of the seizures. Oxaliplatin was discontinued, and the patient was continued on capecitabine monotherapy at 1500 mg twice daily for 14 days in a 21-day cycle. Two months later, he underwent a low anterior resection (LAR).

**Figure 1:**
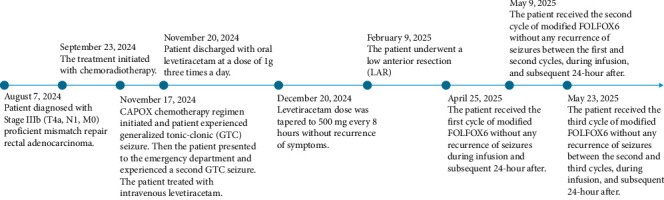
Timeline of the patient's treatment and seizures.

In April 2025, the patient became a candidate for adjuvant chemotherapy with a modified FOLFOX6 regimen. A multidisciplinary team—including an oncologist, a clinical pharmacist, and a neurology specialist—reviewed the case. As the patient had remained seizure-free for over 6 months while receiving levetiracetam, the decision was made to cautiously reconsider oxaliplatin under anticonvulsant therapy. The patient successfully completed three cycles of modified FOLFOX6 without any recurrence of seizures during infusion, between three cycles, and within the subsequent 24-h after the third cycle at the time of discharge (Figure 1).

## 3. Discussion

Oxaliplatin is broadly used for various cancers; however, it is FDA-approved for use in combination with 5-fluorouracil and leucovorin, known as the FOLFOX regimen, in the adjuvant treatment of Stage III colorectal cancer following resection of the primary tumor and for the treatment of metastatic colorectal cancer [1, 3]. Oxaliplatin has a specific pattern of side effects that distinguishes it from other drugs in this class. Hematologic toxicity, gastrointestinal toxicity, and neuropathy are the most common adverse events reported with oxaliplatin [8]. Among neurological complications, acute peripheral neuropathy is the most frequently reported. Rarely, repeated oxaliplatin administration may result in chronic peripheral neuropathy [5]. A rare complication that has been reported with oxaliplatin in case reports worldwide is PRES [6]. This syndrome is characterized by clinical symptoms and radiological findings. Clinical symptoms can be nonspecific including severe headaches, visual disturbances, and seizures to severe symptoms such as coma. Symmetrical and bilateral abnormalities that mainly affect the white matter of the brain are among the pathologic findings seen on typical brain imaging [9]. Seizures following oxaliplatin infusion are rare, with a reported incidence of less than 1% and have been seen in cases where the patient has had a reversible encephalopathy syndrome [10].

In our case, potential causes which could explain the patient's neurological symptoms including brain metastasis, systemic infection, and metabolic disorders were excluded. Hence, chemotherapy seems to be the most likely factor leading to seizures. Oxaliplatin-induced seizures are generally reported as a consequence of PRES which has been mentioned in some case reports with oxaliplatin-based chemotherapy regimens [11]. The findings of the brain MRI in our case were not suggestive of PRES. Additionally, blood pressure of the patient was within normal range, and the patient did not present with visual disturbances, altered mental status, and/or lethargy.

To our knowledge, four cases of oxaliplatin-induced seizures in the absence of PRES were reported [7, 10, 12, 13]. The first case was a 50-year-old man with mixed adenoneuroendocrine carcinoma who developed tonic–clonic seizures lasting 2–3 min during the third cycle of adjuvant chemotherapy with FOLFOX. The patient developed tonic–clonic seizures for the second time after he was rechallenged with the FOLFOX chemotherapy. Thereafter, the patient's chemotherapy regimen was then changed to FOLFIRI (folinic acid, 5-FU, and irinotecan), and the patient did not experience any seizures after receiving five cycles of FOLFIRI; therefore, oxaliplatin was identified as the most suspected cause of the seizures [10]. The second case was a 26-year-old man with metastatic colorectal cancer [12]. The patient presented generalized seizures following cytoreductive surgery alongside hyperthermic intraperitoneal (IP) chemotherapy (HIPEC) with oxaliplatin. The patient had no electrolyte abnormalities, no signs of infection, brain structural alteration, or other metabolic disorder that could possibly explain the seizure. It was concluded that either physiological modifications during HIPEC or IP oxaliplatin may be the possible cause of the seizure. Phenytoin was started to prevent the recurrence of seizures, but was discontinued 10 days later because the patient had no further episodes of seizures. The third patient was a 74-year-old woman with colorectal cancer [13]. A status epilepticus occurred 2 days after oxaliplatin administration. The patient was delirious for 2 days and became conscious with no neurological sequelae 4 days after her seizure. The fourth case was reported by Feki et al. [7]. A 73-year-old woman with Stage IIA gastric cancer developed GTC seizures 4 days after receiving the first cycle of chemotherapy with 5-fluorouracil, leucovorin, oxaliplatin, and docetaxel (FLOT regimen) injection. Seizures lasted less than 2 min with full recovery. Laboratory workup was normal. The patient experienced isolated seizures without any other neurologic accompanying symptoms. She had no electrolyte abnormalities and no signs of infection. Finally, the medical team decided to discontinue chemotherapy and refer the patient for surgery due to the dMMR status. The main characteristics of the reported case of oxaliplatin-induced seizure in the absence of PRES are provided in Table 1.

In our case, the onset of tonic–clonic seizures occurred a few hours after receiving oxaliplatin, while the patient was not receiving any other medication. The seizures occurred at intervals of two and a half hours, and each seizure lasted 1 min. The commonality between all the case reports and our case was the absence of any abnormal laboratory or radiological findings. In all four cases, no evidence of PRES was observed; hence, the hypothesis that PRES is the cause of the seizures induced by oxaliplatin is challenged. Thus, further investigations are necessary to look for mechanisms for the seizures. Of note, only in the case by Rahal et al., rechallenging of oxaliplatin administration was attempted, resulting in another seizure, though it was not stated whether anticonvulsant prophylaxis was used. To our knowledge, this is the first reported case in which the administration of oxaliplatin was successfully rechallenged following an isolated seizure, after receiving an anticonvulsant agent and being seizure-free for an extensive period of time. Although discontinuation seems necessary in cases of oxaliplatin-induced seizure, our case demonstrates that rechallenging the patient with oxaliplatin could be considered in selected patients after thorough risk–benefit assessment by an intradisciplinary medical team.

## 4. Conclusion

This case highlights a rare presentation of oxaliplatin-induced seizure occurring in the absence of PRES or other identifiable metabolic, structural, or infectious causes. Notably, successful rechallenge with oxaliplatin was achieved after a prolonged seizure-free interval and under antiepileptic coverage, suggesting that rechallenging may be considered in selected patients following multidisciplinary evaluation. This case contributes to the limited literature on central neurotoxic effects of oxaliplatin and emphasizes the need for further studies to elucidate the underlying mechanisms and also underscores the importance of individualized, multidisciplinary decision-making in managing rare but serious chemotherapy-related complications.

## Figures and Tables

**Table 1:** Main characteristics of reported cases of oxaliplatin-induced seizure in the absence of PRES.

**Study**	**Malignancy type**	**Chemotherapy regimen**	**Abnormalities in brain CT scan or MRI, EEG, laboratory tests, and vital signs**	**Time of seizure onset**	**Seizure type**	**Intervention**	**Outcome**
Feki et al. [7]	Stage IIA gastric adenocarcinoma, dMMR	FLOT	No	Four days after the first dose	Three separate generalized tonic seizures with ocular revulsion	Chemotherapy was discontinued, and the patient was referred for surgery	N/A
Rahal et al. [10]	MANEC	FOLFOX	No	Third cycle	GTC	Rechallenge of FOLFOX	Incidence of GTC for the second time after the fourth cycle. The patient continued on FOLFIRI chemotherapy without further seizure
Tsukamoto et al. [12]	Metastatic colonic adenocarcinoma, KRAS wild type	CRS with HIPEC	No	Second postoperative day	GTC	Patient started on phenytoin	Phenytoin was discontinued after 10 days due to no recurrence of seizures
Tedjasukmana et al. [13]	Stage III colorectal cancer	FOLFOX	Mild hyponatremia (133 mEq/L)	Two days after the first dose	Generalized status epilepticus	Patient started on diazepam and then phenytoin	Administration of diazepam stopped the seizure. The patient became conscious with no neurological sequelae 4 days after her seizures

Abbreviations: CRS, cytoreductive surgery; CT, computed tomography; dMMR, deficient mismatch repair; FLOT, 5-fluorouracil, leucovorin, oxaliplatin, and docetaxel; FOLFOX, 5-fluorouracil, leucovorin, and oxaliplatin; GTC, generalized tonic–clonic; HIPEC, hyperthermic intraperitoneal chemotherapy; IP, intraperitoneal; MANEC, mixed adenoneuroendocrine carcinoma; MRI, magnetic resonance imaging.

## Data Availability

The data that support the findings of this study are available from the corresponding author upon reasonable request.
